# Examining the Sustained Impact of California’s Tobacco Free for Recovery Initiative: Qualitative Perspectives from Program Directors

**DOI:** 10.1080/10826084.2026.2710339

**Published:** 2026-08-08

**Authors:** Caravella McCuistian, Jennifer T. Le, Elyssa Samayoa, Barbara K. Campbell, Catherine Bonniot, Anna Pagano

**Affiliations:** a Department of Psychiatry and Behavioral Sciences, University of California, San Francisco, San Francisco, California, USA; b Department of Medicine, General Internal Medicine & Geriatrics, Oregon Health and Science University, Portland, Oregon, USA; c Smoking Cessation Leadership Center, University of California, San Francisco, San Francisco, California, USA

**Keywords:** Smoking cessation, substance use treatment, tobacco-free, policy, implementation, sustainability

## Abstract

**Background:**

Smoking prevalence among people in substance use treatment is substantially higher than the national average. Residential substance use treatment represents an opportune time to address this disparity. California launched the Tobacco Free for Recovery Initiative to help residential substance use treatment programs implement tobacco-free grounds policies and increase smoking and other tobacco cessation services. Eleven programs that participated in the initiative were revisited over two years later to examine the sustainability of changes made during the initiative.

**Methods:**

Directors in each of the treatment programs participated in semi-structured interviews. Thematic analysis methods and an implementation science framework were applied to investigate research questions.

**Results:**

Over half of the programs (*n* = 6; 54.5%) remained tobacco free after two years. The other five programs (45.5%) reverted their policies or had never implemented tobacco free grounds. All programs reported offering smoking cessation services at the time of interview, irrespective of tobacco free policies. Directors identified barriers and supports for sustainability and described ongoing needs.

**Conclusions:**

Guided by an implementation framework, results from this study offer recommendations for how to support residential substance use treatment programs in addressing smoking prevalence among clients.

Although smoking prevalence rates have shown promising decreases in the general population (Meza et al., 2023), rates remains high among people with substance use disorder (SUD), whose smoking prevalence rates are three to five times higher than the national average (Han et al., 2022; Saloner et al., 2023). Among individuals accessing SUD treatment, smoking prevalence rates are estimated at 59–77% (Gass et al., 2018; Guydish et al., 2019; Min et al., 2022). Smoking among individuals in SUD treatment has been associated with higher rates of psychiatric symptoms, increased self-report of illicit substance use, and return to substance use following receipt of treatment (Syan et al., 2025; Weinberger et al., 2017). Conversely, smoking cessation is associated with sustained remission of SUD (Parks et al., 2025). SUD treatment seekers who smoke also have more difficulty quitting smoking (Weinberger et al., 2016) and experience higher tobacco-related mortality rates than smokers in the general population (Bandiera et al., 2015). Additionally, smoking rates among SUD counselors are disproportionately high and may contribute to decreased access to smoking cessation services among SUD treatment seekers (Guydish et al., 2022).

While it is apparent that smoking disparately and detrimentally impacts people with SUD, their access to smoking cessation treatment services is limited. Gaps remain in widely integrating evidence-based smoking cessation into primary care (Pipe et al., 2022; VanFrank et al., 2024). People who use substances also experience unique barriers to engagement in primary care (Motavalli et al., 2021; Ross et al., 2015), which may further reduce their access to smoking cessation services. Given this, SUD treatment settings may serve as an important access point for smoking cessation services for people with SUD, given the potential benefit that smoking cessation may have on improving SUD treatment outcomes (Parks et al., 2025).

Smoking cessation services such as tobacco screening and counseling have been increasingly integrated into SUD treatment settings (Abraham et al., 2017; Friedmann et al., 2008; Marynak et al., 2018; Sussman et al., 2026). Despite this, SUD programs experience significant barriers that may limit the impact these services have on client smoking prevalence. SUD treatment programs often have limited resources, staff education, and training in providing smoking cessation services (Pagano, Tajima, et al. 2016). They may also adopt a pro-smoking culture which challenges the adoption and sustainment of cessation services (Eby et al., 2015; Pagano et al. 2016). Moreover, staff attitudes toward smoking cessation could limit the reach of cessation services (Iyahen et al., 2023).

One approach to reducing client smoking prevalence that may address some of these barriers is the implementation of tobacco-free grounds (TFG) policies. TFG policies prohibit tobacco use by staff or clients within program buildings or on program grounds. The implementation of TFG policies has been associated with improved tobacco screening, increased access to smoking cessation services, increased staff education, and even decreased client smoking prevalence (Brown et al., 2012; Campbell et al., 2022, 2025; Correa-Fernández et al., 2019; Drach et al., 2012; Marynak et al., 2018; McCuistian et al., 2022, 2024; Williams et al., 2005). Despite this potential, a recent study conducted by Holt and colleagues revealed that over 69% of residential SUD treatment programs across the United States still permitted smoking on program grounds (Holt et al., 2024), with only nine states implementing statewide policies preventing smoking in SUD treatment programs (Public Health Law Center at Mitchell Hamline School of Law, n.d.).

Within the state of California, over 77% of SUD treatment programs allow smoking on program grounds (Marynak et al., 2018). In an effort to reduce client smoking prevalence, the California Tobacco Control Program launched the Initiative to Reduce Tobacco-Related Disparities at Residential Behavioral Health Facilities (CTCP, 2019), later renamed as the Tobacco Free for Recovery (TFR) Initiative (CTCP, 2020b). The 18-month initiative was designed to support residential SUD programs in developing, implementing, or strengthening cessation services and TFG policies. Results from an evaluation of the TFR Initiative found a decrease in client-reported smoking prevalence and an increase in receipt of Nicotine Replacement Therapy (NRT) (McCuistian et al., 2022, 2024). Despite the promising findings from the TFR Initiative, evidence from other statewide efforts to implement TFG policies highlights concerns regarding sustained impacts. Pagano et al. (2016) found limited change in client smoking prevalence five years after a statewide TFG policy in New York (Pagano et al., 2016). Williams et al. (2005) surveyed thirty-three program directors in New Jersey one year after implementation of a statewide TFG policy and found that 50% had rescinded their policy (Williams et al., 2005). These findings suggest that more evidence is needed on the sustainability and long-term impact of TFG policies in SUD treatment settings. More information about barriers and facilitators to sustainability could inform future implementation strategies for statewide mandates both in California and elsewhere.

The current study aimed to address this gap. Programs that participated in California’s TFR Initiative were revisited two years after the completion of the initiative to participate in the current study. Directors at each program were interviewed. The study aims were to 1) explore any changes in the TFG policy over time, 2) identify any barriers and supports for sustainability of the TFG policies and 3) ascertain any ongoing resources needed to support sustainability.

## Method

### Description of tobacco free for recovery initiative

A total of 17 residential substance use treatment programs were recruited from 2018 to 2021 to participate in the TFR Initiative supported by the California Tobacco Control Program (CTCP, 2019, 2020a, 2020b). Eligibility criteria to participate in the initiative included 1) being a licensed non-profit residential behavioral health facility offering SUD treatment, 2) having a minimum of 15 beds, 3) having been in operation for at least two years, and 4) having no affiliations or contractual relationships with tobacco companies.

Programs voluntarily applied to be considered for the initiative. Those that were selected to participate were supported by the University of California’s Smoking Cessation Leadership Center or SCLC; (Schroeder et al., 2018). Representatives from SCLC coached programs in developing, implementing, or strengthening TFG policies, facilitated programs’ provision of tobacco use assessments and treatment, and fostered development and integration of other wellness activities to support a tobacco-free environment. While most SUD treatment programs participating in the initiative had high rates of cigarette smoking among clients, the Tobacco Free for Recovery Initiative supported programs in reducing all forms of tobacco use. Programs participated in the initiative for 18 months in total, during which time they met monthly (during 30-minute calls) with SCLC representatives to complete needs assessments, create action plans, review current policies, and receive support for new policy implementation. Representatives from each program were also asked to participate in quarterly learning collaborative meetings where attendees could offer guidance, brainstorm solutions to challenges, and motivate one another. Each program also received $36,000 from CTCP to support policy implementation. Of note, while programs were supported in completing the initiative and were encouraged to implement TFG policies, programs faced no consequences if they did not implement or sustain policies. Further details about the initiative are published elsewhere (Campbell et al., 2022, 2025; McCuistian et al., 2022, 2024).

### Participants

Approximately two and a half years after the initiative was completed, the 17 programs that participated in the original initiative were invited to participate in the current study exploring sustainability. Directors from eleven of these programs (64.7% response rate) agreed to participate in the current study and completed in-depth interviews from 2023 to 2025. Data presented in the current study are a part of a larger multi-method study, including survey data from staff and clients (not presented in current manuscript).

### Data collection

After receiving training from a qualitative researcher (AP), the principal investigator (CM) and a research team member (JL) conducted semi-structured interviews through Zoom. The interview guide was informed by the 2009 Consolidated Framework for Implementation Research or CFIR; (Damschroder et al., 2009), which consolidates theories and factors that can influence implementation and sustainability into a single taxonomy of five domains (Intervention Characteristics, Outer Setting, Inner Setting, Individuals, and Implementation Process) and 39 constructs. The CFIR can be used as a framework to guide the qualitative assessment of factors associated with the sustainability of an intervention. When preparing the interview guide, the first author began by identifying several CIFR constructs that would be applicable as barriers or facilitators for sustainability. The first author then drafted interview questions related to these constructs which were then reviewed and edited by all team members. Coauthors CM and JL piloted the interview guide once prior to data collection with a SUD program director uninvolved with the current study. The interview guide was then refined prior to being finalized for use.

Each interview lasted between 45 and 60 minutes and was recorded using Zoom. In addition to participating in the qualitative interview, program directors were asked to complete a short online survey (*via* Qualtrics) to report demographic information and details about their work experience. All participants provided informed consent, and procedures were approved by University of California San Francisco Institutional Review Board (21–35557).

### Data analysis

#### Qualitative interview data

All interviews were transcribed by a professional transcriptionist. Interviews were analyzed using thematic analysis techniques (Boyatzis, 1998). Themes were identified *via* deductive (i.e., guided by CFIR model) and inductive approaches allowing for themes to emerge from the data organically. Data analysis began with three coauthors (CM, ES, JL) repeatedly open coding several transcripts. Identification of salient themes was then conducted with the support of the qualitative expert author (AP), and a codebook was developed. Transcripts and the codebook were then entered into Dedoose, a qualitative data analysis software (Dedoose, n. d.-a). Coding at this stage began in the Dedoose Training Center, which allows coders to build and maintain interrater reliability (Dedoose, n.d.-b). The primary coder (JL) began by coding a transcript using the codebook. The first author (CM) then generated a training session based on the primary coded transcript. The secondary coder (ES) completed the test, which assessed agreement in code application and interrater reliability. Following completion of the test, any discrepant codes were discussed as a group until consensus was reached to clarify code application and reduce further discrepancies. The secondary coder (ES) then coded the transcript fully, discussing any questions with the coding team during weekly meetings. This process was conducted for 36% (*n* = 4) of the 11 transcripts. At the end of these training procedures, the average kappa across all tests was 0.71 (De Vries et al., 2008).

Following training, the 11 transcripts were divided among two coauthors (JL and ES) and coded independently. The two coders met with the first author (CM) weekly and discussed any questions regarding coding application. First author CM then reviewed codes for salient themes based on frequency across the sample ((Buetow, 2010) and identified exemplary quotes.

#### Quantitative survey data

Quantitative data were analyzed *via* descriptive statistics including means and frequencies.

#### Reflexivity statement

Several coauthors on this manuscript held key roles in the evaluation [CM, JL, ES, AP, BC] and delivery [CB] of the original TFR Initiative, which may have influenced data collection and/or interpretation of the qualitative findings.

## Results

### Demographics and other characteristics

The sample for this study included 11 participants from 11 SUD treatment programs (Table 1). Eight participants were directors in their respective programs and completed the interviews independently. A ninth participant was the director of two programs and completed two separate interviews. The tenth participant was the director of a treatment program and was accompanied during the interview by a smoking cessation program manager who assisted in answering questions regarding policies and services offered within the program. Participants’ average age was 43.8 years old (SD = 14.6) and most identified as White (63.6%) and female (72.7%). Most had either a master’s or associate degree (63.7%) and 63.6% had been employed at the participating agency for six years or more. Seven participants (63.7%) reported working generally in the addictions field for over ten years. Many of the program directors identified as being in recovery from SUD (63.6%), though smoking history was less common (only reported by 18.2%). Among the 11 interviews conducted, 63.6% (7 out of 11) of the participants were directors of their respective treatment programs during the TFR Initiative.

### Overview of findings

Our findings are organized below into four categories: 1) current policies and services sustained post initiative, 2) barriers to sustainability, 3) supports to sustainability, and 4) ongoing needs. Relevant themes and exemplary quotes within each category are presented below.

#### Current policies and services sustained after initiative

Approximately 2.5 years after completing the TFR Initiative, 6 of the 11 programs (54.5%) in the current study reported being tobacco free at the time of data collection. Four of the programs (36.3%) had adopted and sustained TFG policies during the Initiative (see “Adopted and Sustained” in quotes below). An additional two programs already had TFG policies at the beginning of the Initiative and maintained them over the 2.5 years (see “Maintained” in quotes below). In total, 6 of the 11 programs (54.5%) in the current study reported being tobacco free at the time of data collection (i.e., four that adopted and sustained policies and two that maintained policies developed prior to the TFR initiative).

Three programs (27.3%) adopted TFG policies during the TFR Initiative but subsequently reverted to allowing smoking on program grounds. Of note, two of these three programs had the same program director. Quotes from these directors are described as “Reverted”.

*“We tried to go completely non-smoking and it wasn’t successful, and so what we did instead is we did a designated [smoking] area close to the edge of the property…it was shortly after [the TFR initiative] that we switched it, just because it was unsuccessful.”* – Program #5 (Reverted)

Finally, two programs (18.2%) participated in the TFR Initiative yet never adopted TFG policies and continued to allow smoking at the time of the current study. However, one program described ongoing efforts to reduce smoking litter to address cigarette sharing. Quotes from these directors are described as “Never Adopted”.

*“And now, we’ve upgraded it to like a contained ashtray… we’ve had issues where they would reach in to pull out cigarette butts… saving the tobacco to smoke and things like that… which is not healthy at all.”* – Program #8 (Never Adopted)

Despite the variability in TFG policies, smoking cessation services were offered at all 11 programs. All programs offered Nicotine Replacement Therapy (NRT) but varied in their method for obtaining this treatment. Several programs encouraged clients to have NRT prescribed by their primary care physicians or supported the clients in calling the statewide Quitline. However, four of the programs emphasized the importance of having NRT on-site for clients, reducing the additional barrier of obtaining a prescription.

*“I decided to just start buying [NRT] myself because I didn’t want the barrier… I want this program to go forward, so we just pay for them now. And if there ever comes a point where we find a way to get them paid for, we will, but I’m not going to let that stop where I want to go.” –* Program #9 (Reverted)

Many programs also offered smoking cessation education for clients, often integrating it into therapy groups focused on wellness topics such as nutrition or physical activity.

*“Our education groups… it’ll be like physical activity one day, smoking cessation one day, nutrition, holistic is a good word for it, so every day of the week they’re getting something.”* - Program #4 (Adopted and Sustained)

Other programs described how broader educational groups focused on relapse prevention were also pertinent to smoking.

*“We don’t have any tobacco cessation groups, but we have a broader focus. We have relapse prevention groups that happen every day… We have recovery support groups that happen essentially every day… So, while we’re not specifically addressing tobacco we are addressing symptoms of an addictive disorder, which covers that.”* – Program #7 (Adopted and Sustained)

Several programs also described the importance of discussing smoking cessation upon intake with clients. Many programs used this discussion as an opportunity to reiterate their TFG policies with clients as they entered the program and “plant the seed” for smoking cessation.

*“As part of the intake questionnaire…individuals are informed that it is a smoke-free place and they are asked if they are smokers and if they are willing to quit. And at that point they are told that if they cannot get a prescription for [NRT], that we will provide that for them, but that they will no longer be able to consume any nicotine products once they are enrolled into our program.*” – Program #6 (Maintained)

Even programs without TFG at the time of data collection reported providing smoking cessation services to clients. All five provided NRT and nearly all (4 out of 5) offered smoking cessation education and offered referrals to the statewide Quitline.

*“We offer [NRT], they do CBT with their therapists…we hook them up through our case management with the no smoking hotline …we do linkages as people want them*.” – Program # 11 (Never Adopted)

#### Barriers to sustainability

Most program directors (*n* = 7) shared that clients experienced significant challenges when trying to stop smoking, which at times threatened client retention. For one program, this challenge became so difficult to overcome that they reverted to allowing smoking. They also reported helping clients purchase cigarettes to retain them in treatment.

*“Ideally, it’s zero tobacco use on the facilities. We’ve tried it several times agencywide…it always results in very bad outcomes, especially with the patients and not just being able to retain them… just a break that you see within the patient of feeling like they have no other escape. So keeping that in mind and coming from a patient centered harm reduction agency, we did have to increase to two smoke breaks… So we did a balancing act… and just to add, we do buy them, we only allow them two packs of cigarettes a week… We send out staff to purchase them.”* Program #10 (Reverted)

In addition to impacting retention, negative impacts of TFG policies on admissions were discussed by several directors.

*“It’s very difficult for clients coming in who are giving up other substances, to give up their cigarettes…it’s often quite a transition for them… And I will say some clients do leave treatment because of it…”* – Program #4 (Adopted and Sustained)

“*Whenever I do client interviews [with] the potential residents, I let them know about the smoking policy and for some of them… they didn’t want to move in based off of their inability to smoke wherever or whenever they wanted.”* – Program #8 (Never Adopted)

Several programs also shared some resistance at the staff level. Program directors reported difficulty encouraging staff to quit smoking, highlighting the “very fine line” between workplace policies versus expecting staff to change behavior during non-work hours (since staff could not come to work smelling like tobacco). One program even highlighted that a staff member who smoked opted to quit their job due to TFG policies.

*“We did have a staff member who got her master’s degree but she said, ‘I really don’t want to work here anymore because you’re non-smoking*’.” – Program #1 (Adopted and Sustained)

Electronic nicotine delivery systems such as “vapes” were also described as presenting challenges for TFG policies. One director was uncertain about how to regulate vape use due to concern that clients could vape non-nicotine substances (e.g., methamphetamine) but also viewing vapes as personal property of the client.

*“So vapes was a very gray area, like what do we allow them to keep in their belongings …we tried for a little bit like, well, you can take it with you on pass, it’s still your belonging …we’re not going to unlawfully withhold their stuff… vapes are unique in that they can smoke whatever in it… a vape is a tricky thing. So now I believe our current policy is just none of it, you just can’t, no vapes, no chambers.”* – Program #4 (Adopted and Sustained)

Many of the programs reported that they approached smoking cessation and TFG policies through a harm reduction framework. Several directors from programs that either reverted or never adopted TFG policies viewed allowing smoking as harm reduction since it helped retain clients in treatment. One director that adopted and sustained TFG policies even opted to allow smoking in one of their several programs to accommodate individuals who did not wish to quit smoking while in treatment. Another described how allowing clients to smoke was seen as a support for emotional regulation during times of stress; they discussed program-level efforts to rebrand smoke breaks to “mental health breaks” during which clients were allowed to smoke.

*“So they aren’t necessarily smoking breaks, they’re called mental health breaks, but it’s during that time that they are allowed to smoke. So the verbiage has changed…just to encourage harm reduction.”* – Program # 10 (Reverted)

#### Supports for sustainability

When discussing supports for sustaining changes made during the initiative, directors often described their approach to smoking cessation as “holistic”. They reported integrating smoking cessation services into existing SUD services to help clients reduce their overall substance use.

*“The ones that are interested in smoking cessation… they join the health and wellness group…and they get mp3 players with meditations on them…if they’re doing smoking cessation, if they’re doing gambling, if they’re doing sleep, whatever…and they’re getting healthier and eating vitamins and running and they’re quitting smoking… we have had quite a few people quit smoking because of [this] group. This group is very attractive.”* – Program #9 (Reverted)

Given the high rates of staff smoking in residential substance use treatment (Guydish et al., 2007), several programs recognized the importance of having staff be “role models” for TFG policies by not smoking on site or showing signs of smoking behavior in front of clients.

*“We don’t formally have a policy like we do for the clients for our staff but we certainly sort of peripherally have built a culture on our team…a culture of wellness… so I had a staff member be like, “you don’t want to be a hypocrite”… and so I don’t smoke…I don’t do certain things because we’re trying to tell the clients not to do certain things.”* – Program #4 (Adopted and Sustained)

Other participants described having specific staff ensure that policies were implemented and services were maintained. Often these individuals were the program directors themselves, who described the importance of remaining persistent with the policies. One director described persisting with TFG during an initial census drop shortly after implementation which eventually returned to normal.

*“We went through that whole process and we never went back. We still have clients that show up for residential that are like, “Oh, I didn’t really understand you’re smoke free,” and so they leave. And when we first went through it… some of my staff said, “We’re not able to get people in, why don’t we go back to a few times a day smoking?” No, we’re not going back. When we made the decision, we’re not going to go back. And now we’re full.”* – Program #1 (Adopted and Sustained)

Three of the programs also described receiving support from their county, often in the form of financial support to pay for NRT. One program described how their county implemented a larger policy requiring tobacco-free grounds in behavioral health settings, which supported their ability to sustain a TFG policy. Without this county support, the director questioned whether they could remain tobacco free.

Participant: *“Our local county Behavioral Health Division… implemented [it] into all of the providers’ contracts to become non-smoking facilities.”*

Interviewer: “*Do you think things would be different if at the program level people could decide if they were non-smoking or not, like maybe some were and some weren’t?”*

Participant: *“…I think that if there were another program that also enrolled folks that were in our population that allowed smoking, we’d have a hard time filling the beds.”* - Program #6 (Maintained)

#### Ongoing needs

Nearly all directors identified a need for more resources to sustain TFG policies and smoking cessation services including affordable access to NRT, comprehensive screening tools for nicotine dependence, interactive resources for client education, and contingency management. Two directors also mentioned challenges with reimbursement which limited their ability to provide smoking cessation services.

“*It’s really challenging when you are doing an assessment, a client clearly has a tobacco use disorder and you know that’s not something you can treat directly…we can’t really touch that. So I guess this is more of a policy level change…because if you look at the regulations it says that tobacco use disorder specifically cannot be treated in a Drug Medicaid program.”* – Program #7 (Adopted and Sustained)

Another director described the potential benefit of an upcoming statewide policy to prevent smoking in residential treatment programs across the state. They indicated that such a policy could have effects beyond SUD treatment by improving community health and reducing the financial burden of smoking (e.g., increased healthcare expenditures for smoking-related illnesses).

*“SAMHSA, their definition for recovery includes health and wellness…non-smoking is health and wellness…we never want to tell anybody what to do but why should money go towards letting them smoke when that’s going to cause more health damage and help them relapse faster. And then getting them sicker, the whole community sicker, and more money spent.”* – Program #1 (Adopted and Sustained)

## Discussion

Despite recent declines in smoking in the general population (Centers for Disease Control & Prevention, 2024), smoking rates remain high among people in residential substance use treatment (Gass et al., 2018; Guydish et al., 2019; Min et al., 2022). To reduce these disparities, California launched the TFR Initiative. The current study explored the sustainability of the initiative two years following completion.

Results from the current study show that most programs either maintained previously developed TFG policies or sustained policies that they adopted during the initiative (54.5%). However, five programs (45.5%) faced significant barriers that resulted in them reverting to allowing smoking and/or deciding not to implement TFG. This proportion is similar to a previous study finding that 50% of programs had rescinded TFG policies in New Jersey one year after a statewide policy (Williams et al., 2005).

### Barriers to sustainability

Programs faced several barriers that threatened the sustainability of the Tobacco Free for Recovery Initiative (Table 2). For example, several barriers related to the financial cost of maintaining the initiative. Some programs faced reimbursement-related challenges under the current funding policies within Medicaid, and one director even described having to purchase NRT out of their own pocket. Several other directors described challenges with maintaining client census (at least initially) following implementation of TFG policies, which could impact program revenue. These results reiterate that while SUD treatment may be well-positioned for offering smoking cessation services (Baca & Yahne, 2009), strategies for overcoming financial barriers should be considered throughout implementation.

Another barrier to sustainability was related to cultural norms within some SUD treatment programs. Several directors mentioned that TFG policies were not aligned with harm reduction frameworks. Other qualitative studies have reported a common view among SUD treatment providers that TFG policies may negatively impact recovery (Iyahen et al., 2023), despite evidence that smoking cessation services are associated with improved substance use outcomes (McKelvey et al., 2017; Parks et al., 2025). However, the contextualization of these concerns *via* a harm reduction framework may be novel. Several participants described the nuanced relationship between attempting to offer smoking cessation services (including implementing TFG policies) while providing client-centered services (e.g., offering smoke breaks for “mental health breaks”). Harm reduction and smoking cessation are not inherently opposed and can be leveraged together to support the overall health and well-being of clients. There have been efforts to reconcile smoking cessation with harm reduction principles, such as recommending the use of e-cigarettes to quit smoking (Warner, 2019). However, this approach may not be appropriate for residential SUD treatment given the challenges mentioned by directors in regulating their use. Participants in the current study identified methods such as encouraging clients to reduce number of cigarettes and offering NRT as harm reduction approaches more aligned with program culture.

### Supports for sustainability

Despite report of several barriers, directors also highlighted supports for sustaining the TFR initiative (Table 2). One support mentioned by several directors was intervention adaptability. For example, several directors described how they were able to integrate smoking cessation services into current programs of care, such as adding smoking cessation education into an established class on health and wellness. Some directors also described adopting their practices to make NRT more affordable, such as connecting clients to their primary care physicians at intake since insurance would cover NRT if prescribed.

Several directors described programs having a holistic culture that facilitated sustainment of TFG policies. Programs offered auxiliary services to clients in addition to SUD treatment such as nutrition and physical activity counseling, which were additional topics highlighted in the TFR Initiative. Smoking cessation services easily fit within this structure. Promoting a holistic culture seemed to impact the behavior of staff, as directors mentioned staff wanted to be “role models” and avoid showing signs of smoking to clients. This culture shift may be especially helpful in SUD treatment programs, where staff smoking is higher than the national average (Guydish et al., 2007) and concurrent staff and client smoking is associated with increased client smoking prevalence and decreased rates of smoking cessation services (Guydish et al., 2022). Thus, a wellness focus may be viewed as an adaptable component of the TFR initiative that fostered a TFG sustainment culture in programs that utilized it.

Another important sustainability facilitator reported by study participants was county level support in the form of funding for NRT or county-wide policies preventing smoking. A county-wide policy mandating TFG was seen as a facilitator for one program. Without this mandate, the program questioned whether they would be able to sustain a TFG policy. Given the concerns participants raised about client census and retention due to TFG policies, county or statewide policies mandating TFG may warrant further consideration. However, the sustainability of these statewide policies should be examined further.

While several programs highlighted financial limitations to sustaining smoking cessation services, other programs adapted their services to circumvent it, suggesting programs may benefit from education on how to integrate smoking cessation counseling within their current fundable services. County-wide support to offset any financial barriers may also be useful.

### Study limitations and future directions

The current study is not without limitation. The sample is small and represents only 11 of the 17 programs (64.7%) that participated in the original TFR initiative, as several programs did not consent to participate in this additional study exploring sustainability. These decisions may have been impacted by changing priorities for the programs, high staff turnover, or other competing priorities (e.g., COVID) that reduced the programs’ ability to engage in research at the time of the study. Thus, the findings presented in the current study may not fully represent all the participants in the TFR Initiative. Furthermore, programs were limited to California, which also limits generalizability. The authors on the current study were also involved with delivering and evaluating the TFR Initiative, which could have introduced bias both in the program director’s participation in the interviews and the interpretation of the results. Finally, the findings in this manuscript are limited to interview data from directors only and do not represent any findings from other key stakeholders including staff and clients.

Despite limitations, study findings hold potential for public health impact. Smoking prevalence among people with SUD is nearly five times that of the national average (Gass et al., 2018; Guydish et al., 2019; Min et al., 2022) and this group faces unique barriers to accessing primary care (Motavalli et al., 2021; Ross et al., 2015), which could significantly limit access to smoking cessation services. SUD treatment centers may be well-suited to address smoking cessation, but also face significant barriers. The Tobacco Free for Recovery Initiative supported programs in overcoming such barriers and implementing a tobacco free grounds policy to reduce smoking prevalence.

Results suggest that several residential SUD programs were able develop and sustain TFG policies when they had access to financial resources, promoted culture change among clients and staff, and integrated smoking cessation into the treatment services they already provide. Some programs found that TFG policies impacted client retention because they were not aligned with harm reduction frameworks, and some opted to rescind or not adopt TFG policies. However, all programs reported increased smoking cessation services, which suggests that efforts such as the Tobacco-Free for Recovery Initiative may be helpful in increasing and sustaining client access to smoking cessation, even in the absence TFG policies. Additional supports such as increased technical assistance or financial support could be considered to further support the sustainability of this and similar initiatives.

While programs in the current study implemented tobacco free grounds and smoking cessation services in the absence of any statewide policies, a recent assembly bill (AB 541) was passed in California that may encourage smoking cessation services in SUD treatment programs (Assembly Bill 541: Tobacco Assessment, 2021). While this bill requires licensed and certified SUD treatment programs to assess for tobacco use disorder and either provide or refer patients to tobacco cessation treatment, it notably does not require programs to implement tobacco free grounds. Programs are also not provided with additional education or resources to implement smoking cessation through this legislation. Initiatives such as Tobacco Free for Recovery could help fill these gaps as legislative changes related to smoking cessation in SUD treatment programs are implemented.

## Figures and Tables

**Table 1.** Demographics and other characteristics (*n* = 11).

Demographic variable	N (%) or mean (SD)
**Age**	43.8 (14.6)
**Gender** Male	3 (27.3%)
Female	8 (72.7%)
**Race** White	7 (63.6%)
Black	1 (9.09%)
Asian	1 (9.09%)
Hispanic/Latino	2 (18.2%)
**Education** Master’s	5 (45.5%)
Associate	2 (18.2%)
Some College, No Degree	2 (18.2%)
High School Diploma/Equivalent	1 (9.1%)
Other	1 (9.1%)
**In Recovery from SUD** Yes	7 (63.6%)
No	3 (27.3%)
Decline to answer	1 (9.1%)
**Smoking Status** Never Smoked	9 (81.8%)
Former Smoker	2 (18.2%)
**Years at Agency** 1–5 years	4 (36.4%)
6–10 years	4 (36.4%)
11–15 years	2 (18.2%)
Over 15 years	1 (9.1%)
**Years in Addiction Field** 1–5 years	1 (9.1%)
6–10 years	3 (27.3%)
11–15 years	3 (27.3%)
Over 15 years	4 (36.4%)

**Table 2.** Overview of facilitators and barriers of sustaining the tobacco free for recovery Initiative.

Barriers	Facilitators
• Financial consequences of initiative	• Intervention adaptability
• Misalignment with program culture	• Adopting a holistic approach to SUD treatment that includes wellness
	• County level support
	• Integration of smoking cessation into fundable services

## Data Availability

The participants of this study did not give written consent for their data to be shared publicly, so due to the sensitive nature of the research supporting data is not available.
